# Cognitive Interpersonal Model for Anorexia Nervosa Revisited: The Perpetuating Factors that Contribute to the Development of the Severe and Enduring Illness

**DOI:** 10.3390/jcm9030630

**Published:** 2020-02-27

**Authors:** Janet Treasure, Daniel Willmott, Suman Ambwani, Valentina Cardi, Danielle Clark Bryan, Katie Rowlands, Ulrike Schmidt

**Affiliations:** 1 Section of Eating Disorders, Department of Psychological Medicine, Institute of Psychiatry, Psychology and Neuroscience, King’s College London, SE5 8AF London, UK; janet.treasure@kcl.ac.uk (J.T.); valentina.cardi@kcl.ac.uk (V.C.); danielle.clarkbryan@kcl.ac.uk (D.C.B.); katie.rowlands@kcl.ac.uk (K.R.); ulrike.schmidt@kcl.ac.uk (U.S.); 2 Department of Psychology, Dickinson College, Carlisle, PA17013, USA; ambwanis@dickinson.edu

**Keywords:** anorexia nervosa, cognitive interpersonal model, severe enduring

## Abstract

The cognitive interpersonal model was outlined initially in 2006 in a paper describing the valued and visible aspects of anorexia nervosa (Schmidt and Treasure, 2006). In 2013, we summarised many of the cognitive and emotional traits underpinning the model (Treasure and Schmidt, 2013). In this paper, we describe in more detail the perpetuating aspects of the model, which include the inter- and intrapersonal related consequences of isolation, depression, and chronic stress that accumulate in the severe and enduring stage of the illness. Since we developed the model, we have been using it to frame research and development at the Maudsley. We have developed and tested interventions for both patients and close others, refining the model through iterative cycles of model/intervention development in line with the Medical Research Council (MRC) framework for complex interventions. For example, we have defined the consequences of living with the illness on close others (including medical professionals) and characterised the intense emotional reactions and behaviours that follow. For the individual with an eating disorder, these counter-reactions can allow the eating disorder to become entrenched. In addition, the consequent chronic stress from starvation and social pain set in motion processes such as depression, neuroprogression, and neuroadaptation. Thus, anorexia nervosa develops a life of its own that is resistant to treatment. In this paper, we describe the underpinnings of the model and how this can be targeted into treatment.

## 1. Introduction

The Medical Research Council has described a framework for the process of development of complex interventions [1]. This involves a procedure in which treatments targeting risk or maintaining factors for an illness are fine-tuned following feedback from proof of concept, feasibility, and pilot studies, including both quantitative and qualitative outcomes. Furthermore, within UK health care research conducted in the National Health Service (NHS), a strong emphasis is placed upon the involvement of patients, carers, and the public to co-design interventions and services. We have used this combined approach to design interventions and services for people with anorexia nervosa (AN).

**Figure 1:**
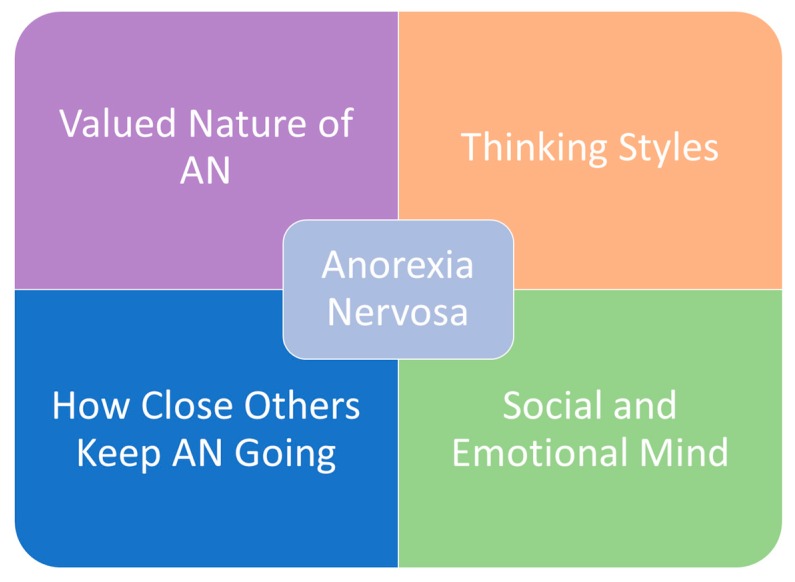
The cognitive–interpersonal maintenance model of anorexia nervosa.

The first iteration of the cognitive interpersonal model for AN outlined core valued and visible maintaining factors of the illness (Figure 1) [2]; the visible aspects of the disorder elicit deep concern from close others, which is in stark contrast to the egosyntonic nature of the illness, where the sufferer values certain aspects of the illness and does not identify themselves as ‘sick’. In the next iteration of the model, we outlined the underpinning neuroscience, the interaction between vulnerability traits and eating disorder behaviours, and how this serves to strengthen the hold of the disorder, allowing it over time to develop a life of its own [3]. Indeed, individuals with a persistent form of the illness often state that they want to change but cannot do so. Examples of interactions between vulnerability traits and eating disorder behaviours include personality traits such as conscientiousness and introversion [4], which can predispose to disordered eating [5] and allow eating disorder habits to become fixed. Furthermore, recent genetic findings reported on correlations between polygenic risk scores, not only with other “brain” disorders, but also with “metabolic” traits related to growth, size, lipids, and insulin sensitivity [6]. Thus, a possible hypothesis is that homeostatic forces maintaining energy balance might be weaker in terms of increasing appetite and stronger in terms of a foraging or over active response to “food shortage”. This argument has been developed in more detail in an evolutionary-based model of AN and might benefit from further exploration [7].

## 2. Staging Models of Anorexia Nervosa: the Enhanced Cognitive Interpersonal Maintenance Model

**Figure 2:**
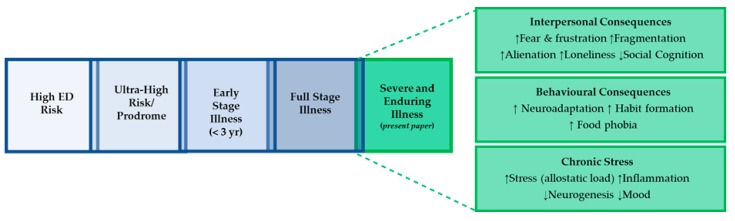
Severe and enduring anorexia nervosa: a maintenance model.

This next iteration of the model describes in more detail the development of the severe and enduring form of AN [8]. Current diagnostic procedures do not account for clinical features such as illness trajectory, severity, and co-morbidity, which may impact on the effectiveness of current treatments. The maintenance model of severe and enduring AN consists of three components (Figure 2). The first domain includes the interpersonal consequences of AN. These arise in part because of the impact of chronic starvation on social cognition and the reaction of others to the illness. The next domain relates to the behavioural consequences of AN. Neuroadaptation and habits develop as a consequence of repeated behaviours. Moreover, feeding experiences can increase aversive learning towards food. The third domain relates to the chronic stress response, which may relate to a genetic predisposition to susceptibility, early adversity, or be a secondary response to chronic starvation. 

In this paper, we consider in more depth these three domains that contribute to the development of the severe enduring form of illness. 

## 3. Social Risk and Maintaining Factors

Difficulties with interpersonal relationships predispose to the development of AN and are also secondary consequences of the disorder, contributing to the perseverance of the illness. 

Social anxiety is a common precursor of eating disorders [9]. In some cases, this is part of the autistic spectrum profile of symptoms that has been observed [10,11], and problems with social cognition may form part of the genetic liability. For example, the offspring of mothers with a history of an eating disorder had anomalies in social cognition during childhood [12]. This familial association suggests that problems in social cognition may be an endophenotype that increases the risk of developing an ED. In addition, adverse social experiences during development such as childhood maltreatment [13], insecure attachment [14], or teasing, bullying, or social exclusion [15] can shape interpersonal relationships and disrupt normal identity development, potentially by increasing the salience of competitive patterns of interaction that can fuel striving for perfection and greater internalisation of beauty ideals as a standard to define the self [16].

Furthermore, people with AN are more likely to report early maladaptive schema (pervasive negative themes regarding the self and one’s relationship with others [17]) and difficulties in emotion regulation, with a greater use of dysfunctional interpersonal emotion regulation strategies [18,19]. Problems in emotion regulation may play a key role in maintaining the symptoms of AN [20]. Thus, an interaction between social vulnerability traits, risks within the interpersonal environment, and dysfunctional emotion processing can predispose to the development of AN, a “social–emotional disorder” [21]. 

Problems in social cognition also can arise as secondary consequences of starvation. Anomalies in social cognition are most pronounced in the acute phase of illness and are less marked after recovery [22]. The anomalies include deficits in nonverbal emotional expression [23,24], an inability to provide a spontaneous social narrative and to identify the social salience of stimuli [25], a sensitivity to threat [26], to social comparison [27], misalignments in social reciprocity [28], and the avoidance or suppression of emotions, particularly to avoid conflict [19]. Impairments in nonverbal communication and a lack of reciprocation of warmth elicits the “uncanny valley” aversive response in others [29,30,31]. This can lead to social exclusion and isolation, which is a defining facet of the illness [32]. 

### 3.1. Interpersonal Reactions to Living with Anorexia Nervosa

A mental health professional with lived experience of AN used one word to epitomize the illness: “isolation” [32]. However, family members are usually closely involved in the evolution of AN, which commonly has its onset in early adolescence. Close others can encourage help seeking in the early phase of the illness, which in turn may have a favourable effect on the prognosis. Nevertheless, the individual’s resistance to the need for treatment can make negotiating this transition difficult. The disruption of family functioning, work, and social adjustment varies accordingly with the developmental age, mood, and other clinical features. Many patients with a chronic form of illness remain dependent on their families or the state during their lifetime [33]. 

The anxiety engendered by the overt “ill state” and the frustration caused by the resistance to implement the obvious solution “to eat” fuels a profound and mixed emotional reaction in both professionals (nurses on inpatient units) [34] and families [35]. Lack of insight into the illness is particularly pronounced in restrictive AN and persists during and after treatment [36,37]. Critical, hostile, overprotective, and controlling behaviours can develop as counter reactions [38,39]. Family members may appease and collude with eating disorder behaviours, becoming organised around eating disorder rules (accommodating to the illness) and ignoring or covering up for the negative consequences of the behaviours (inadvertently enabling the illness) [40].

These diverse reactions can cause divisions amongst family members. Some family members shoulder an overly high burden, whilst others become disempowered and disengaged. Aspects of the caregiving experience are summarised in systematic reviews [35]. High perceived burden and low caregiving efficacy are common and are associated with clinical levels of depression and anxiety. In addition, the societal reaction to the illness (including interactions with services) can be problematic.

Carers’ own practical and emotional reactions to the illness can cause harm to the family unit as relationship ruptures occur in response to accommodating and enabling behaviours [41] or divergent forms of reactive expressed emotion develop. Siblings can be drawn into caregiving, develop their own problems, or leave home prematurely as their needs may be neglected [42,43]. Thus, a vicious circle of escalating interpersonal stress can develop. 

The protracted course of the illness and the entanglement between eating and social function means that close others have the potential to remediate these difficulties. Therefore, it is important that carers and close others are involved across the lifespan and not excluded from the treatment process. Clinicians explain this exclusion through concerns around confidentiality, but this is considered a misinterpretation of the law [44].

### 3.2. Targeting Stressful Interpersonal Reactions

A hypothesis that follows from the cognitive interpersonal model is that the toxic consequences that accrue from the isolation and loneliness of an eating disorder can be ameliorated by skills training for family members or close others across the lifespan [45]. Improving the interpersonal environment often begins at home. Close others are highly motivated to help but are often uncertain of what they can do. Materials coproduced with carers and patients have been developed to illustrate these problems [39] and provide a curriculum for workshops to help carers provide effective support. 

These materials explain how the patients’ primary and secondary difficulties in social cognition make relationships difficult. For example, the reduced facial expressivity of eating disorder patients [23,24] makes it hard for others to appreciate their level of terror or show appreciation of the carers’ distress. AN patients appear to be impervious to the impact of their behaviours on other people. This lack of concern for others appears in the domain of caregiving when others are distressed at their lack of insight and unwillingness to accept the sick role. It also can be evident in treatment facilities where overt demonstration and discussion of eating disorder behaviours can trigger distress in their peers. 

The three basic elements of carer skill-sharing interventions include:

(1) Psychoeducation about the illness and framing patient presentation within the cognitive–interpersonal model, highlighting predisposing, precipitating, and perpetuating factors.

(2) Information about how carers might be able to moderate and mould the energy and insight from their own emotional reactions to foster a calm, compassionate, and collaborative context (a life skill that they can role model to the person with the eating disorder). 

(3) Introduction to effective support and behaviour change skills such as motivational interviewing, the use of risk-taking experiments, and exposure to fear [46]. 

Interventions based on this model have been delivered in a range of different formats including workshops and guided self-help interventions with individual or group support. An improved understanding about the illness reduces carer distress and burden [35,45,47,48]. Several studies have found that augmenting treatment with these resources for carers reduces both carer and service burden [49,50,51]. Often this has involved a ‘train the trainer’ approach whereby carers and patients deliver these interventions. Pépin and King [52] tested the workshops in a pilot study in Australia, finding participation led to reductions in carer distress and burden and helped them to modify their emotional reactions to the illness. Psychoeducational carer workshops reduce carer psychological distress and burden, yet when collaborative carer skills are taught as well, there are secondary effects on alleviating patients’ anxiety and depression [53].

Treatment for individuals with AN based on the cognitive interpersonal model, the Maudsley Model of Anorexia Treatment for Adults (MANTRA) [54,55], also includes a section for carers. Carers are encouraged to cultivate their own emotional and social intelligence by examining the relevant modules for patients so that they can role model these skills to their loved ones. It is helpful if both parties externalise the illness and understand how the AN voice can trap everyone into spirals of confusion. A compassionate, mutually supportive family environment is essential as it is inevitable that miscues and mistakes will happen, but these can be reframed as being valuable opportunities of learning.

## 4. Behavioural Consequences of Anorexia Nervosa

The repeated patterns of behaviour that underpin the development of AN, such as avoiding high caloric, highly palatable foods, becomes habitual. Initially, these behaviours are initiated to achieve a specific goal and are reinforced as they are positively rewarding. With daily repetition, the actions become automatic and entrenched until they are activated with no conscious effort in response to a stimulus or cue [56]. A recent cognitive neuroscience model of AN proposes that habitual behaviours are mediated within dorsal frontostriatal circuits [57,58,59]. In line with this, techniques from habit reversal therapy with inpatients led to significant reductions in eating disorder symptoms and self-reported habit strengths [60]. These preliminary findings could be used to augment or guide developments in treatment for those with severe and enduring difficulties.

In addition, fear and/or disgust responses to food represents a key challenge to weight restoration [61]. People with AN show reduced extinction of fear learning but increased fear generalisation and reinstatement, which may contribute to the strength of these fear learning networks [62]. These fears may originate from adverse experiences relating to food and body shape during sensitive periods of development [63]. Furthermore, these could be iatrogenically aggravated by coercive treatment experiences such as nasogastric feeding under restraint. A qualitative study with nurses identified that power battles over ‘punitive’ feeding causes patients great anguish and can rupture the therapeutic alliance [64]. These interactions may be overlooked as a complicating feature within family-based treatment [65]. Interestingly, in adolescence, reduced extinction of fears appears to be more pronounced with co-morbid depression [66].

Exposure is the standard treatment for fear learning. The underlying mechanism involves developing new learning to the threatening stimulus (as opposed to habituation to anxiety) and updating the fear pathways in the brain [67,68]. There are some caveats in applying this treatment to AN. First, one of the feared consequences, weight gain, is an expected and necessary consequence of refeeding. Furthermore, with fluid fluxes and other aspects of metabolic instability during this phase, a “lack of control of weight” can maintain AN cognitions. Moreover, it is probable that neurogenesis is reduced as a sequelae of the chronic stress of AN, reducing the effectiveness of exposure. A variety of techniques have been used to facilitate this learning. These include positive mood induction [69], the addition of cycloserine to consolidate learning [70,71], and virtual reality [72].

Other training approaches developed to target risk factors show some promise in proof-of-concept studies. These include cognitive remediation approaches that focus on cultivating a flexible, big picture style of thinking [73], cognitive skills training for social avoidance [74], and go/no-go inhibition training [75], which may be of value for the binge–purge form of the illness. Food-related attention bias modification training is also being investigated as an add-on intervention for AN, following directions from anxiety disorder research [76,77].

## 5. Chronic Stress Response: Co-Morbidity with Depression and Anxiety

Another maintaining factor in the model is the impact of chronic starvation leading to anhedonia and accentuating existing co-morbidities. Emotional distress is common as both a predisposition and a secondary consequence that perpetuates the illness [20,78,79]. This emotional distress may occur due to a shared genetic predisposition [6]; developmental trauma [80], with emotional abuse (e.g., bullying) playing a central role [81]; or as a feature of starvation [82].

Depressive symptomatology may follow a persistent trajectory over the lifetime, independently from eating disorder symptoms [83,84]. Approximately 50% of patients with AN report a minimum of one episode of a mood disorder during their lifetime [85,86]. Depression, anxiety, and interpersonal sensitivity are central nodes in a network analysis of symptoms [87] with key bridging roles [81]. The symptom profile differs somewhat from that of unipolar depression in that somatic features such as loss of energy and reduced activation are less pronounced, whereas cognitive features such as negative views of the self are higher [88]. Indeed, the chance of recovery is reduced in people who developed AN after they had experienced depression [89]. In addition, the long-term outcomes of patients with AN complicated with mood comorbidity were reduced [90]. Consequently, psychological well-being, positive supportive relationships, hope, identity, meaning, and purpose are important markers of recovery [91,92].

Anxiety traits are also linked to eating disorders. Obsessive–compulsive personality traits, obsessive–compulsive disorders, and anxiety [9,93,94] often present in childhood prior to the onset of AN. In addition, there is a strong familial association with these traits [95,96]. The strongest polygenic risk correlations from genome wide association studies (GWAS) of AN is shared with OCD [97], whereas correlations with neuroticism, depression, and anxiety are half the size [6]. In a network analysis of eating disorder and anxiety traits, avoidance of social eating was the strongest bridge node, with eating symptoms and low self-confidence being the strongest bridge node with anxiety traits [98].

In a longitudinal study, the neural correlates of disordered eating and obsessive–compulsive disorder at age 14 included an increase in grey matter volume in the orbitofrontal cortex, the right dorsolateral prefrontal cortex, and the ventral striatum [99]. However, people with a chronic illness had a decrease in grey matter in the cerebellum and the mesencephalon, specifically the substantia nigra and ventral tegmental area [100]. Hippocampal volume is particularly reduced [101,102,103] which is associated with anxiety and depression [102] and correlated with raised cortisol [104]. Patients in the later stage of AN have increased ventral striatal activation to body image cues than patients in the early stage of illness [105,106].

The chronic stress profile associated with enduring AN [107] is similar to that found in treatment resistant depression [108] and with the ‘ecophenotype’ associated with childhood maltreatment [109]. This chronic stress response includes hyperactivity of the hypothalamic pituitary adrenal (HPA) axis [101,110,111,112], activation of the immune system [113,114], and anomalies in the microbiome [115,116]. There is a widespread reduction in brain volume, which is possibly secondary to reduced neurogenesis [117], which may be the most marked in the hippocampus [118] and associated with raised cortisol levels [104]. This broad stress response reduces resilience [119] and may be accompanied by reduced mesolimbic dopamine function, anhedonia, and lack of motivation [120]. Thus, a sizeable proportion of patients could be classified as fulfilling the criteria of treatment-resistant depression.

### Treatment-Resistant Depression and other Co-Morbidities as Maintaining Factors: Implications for Treatment

Treatments targeting nutritional recovery are less effective for improving mood [49,50,79,121,122,123]. Therefore, it is possible that strategies used for treatment-resistant depression might be of benefit for people with severe enduring AN. Proof-of-concept studies of neuromodulation such as deep brain stimulation [124] and repetitive transcranial magnetic stimulation have shown potential [125,126,127]. Interestingly, the trajectory of symptom change following these neuromodulation techniques shows differences to that found with more traditional eating disorder treatment, in that an improvement in mood is the main initial effect, whereas improvements in eating disorder psychopathology follow later [125,126,128].

Interest in pharmacological approaches is starting to increase, as summarised in a recent review [129]. There is mixed evidence for the use of antipsychotic medications to treat AN [130], although a recent multicenter outpatient study of olanzapine found small positive effects on agitation and weight gain (albeit with no difference in rates of hospitalisation) [131]. The field may need to consider using drugs that have been shown to have benefit in OCD. For example, high doses of Selective Serotonin Reuptake Inhibitors (SSRIs), exposure, and response treatment are effective for people in the later stages of OCD [132], which has also been recently conceptualized within a staging framework [133]. In addition, there is interest in pharmacological approaches that are being used for treatment-resistant depression, such as ketamine and psilocybin [134]. Interestingly, ketamine showed some promise in a small series of studies in the 1990s [135]. A pilot study of the effects of ketamine on mood and eating disorder cognitions in people with severe and enduring AN is registered on the Australia New Zealand Clinical Trial Registry (anzctr.org.au, ACTRN12618001393246p) and additionally, a psilocybin trial for AN is currently registered (ClinicalTrials.gov, NCT04052568). Therefore, we echo the call by Franko and colleagues [90] to consider using a combined approach to target both mood and eating disorder symptoms, with clear characterization of the patient group.

Although not the topic of this paper, given the similarities between staging models for other psychiatric disorders [8], it may be of interest to look at the other end of the trajectory, the ultra-high risk (UHR) group, and consider whether there might be interventions that could be matched to this stage and provide primary or secondary prevention approaches. The first step is to define the UHR phenotype, which may include traits such as behavioural inhibition, perfectionism, early symptoms of OCD, or social–emotional difficulties.

## 6. Conclusions

In this latest iteration of the cognitive interpersonal model, we have described treatments targeting modifiable elements. Increasing social connection through encouraging a collaborative inclusive approach towards recovery, by increasing the knowledge and skills of family members, decreases the need for high-intensity care (day or inpatient) and reduces carer burden and patient isolation [49,50,51,136]. Treatment using MANTRA focuses on both inter and intrapersonal model elements [55,56]. Augmenting treatment through digital technology is showing potential [137]. Finally, we are borrowing approaches that show promise in other domains of psychiatry in which a chronic stress response has led to the illness having a life of its own. Neuromodulation techniques are encouraging, and new pharmacological approaches are in progress. Services may need to implement broader clinical assessment skills to more accurately stage the illness to personalise treatments to match patient’s needs and preferences.

However, it is too early for this model to be ossified. The field of eating disorders has been starved of resources for research, and so the evidence is limited. Nevertheless, we think that by using this framework in order to identify targets and by using high-quality randomised controlled trials, we will be able to develop more effective treatments through an iterative process.
